# *Optix* defines a neuroepithelial compartment in the optic lobe of the *Drosophila* brain

**DOI:** 10.1186/1749-8104-9-18

**Published:** 2014-07-29

**Authors:** Katrina S Gold, Andrea H Brand

**Affiliations:** 1 The Gurdon Institute and Department of Physiology, Development & Neuroscience, University of Cambridge, Tennis Court Road, Cambridge CB2 1QN, UK; 2 Present address: Department of Cell and Tissue Biology and Eli and Edythe Broad Center of Regeneration Medicine and Stem Cell Research, University of California San Francisco, 35 Medical Center Way, San Francisco, CA 94143-0669, USA

**Keywords:** Neuroepithelium, Stem cell, Adhesion, Compartment, Optix, Six, Visual system, Brain

## Abstract

**Background:**

During early brain development, the organisation of neural progenitors into a neuroepithelial sheet maintains tissue integrity during growth. Neuroepithelial cohesion and patterning is essential for orderly proliferation and neural fate specification. Neuroepithelia are regionalised by the expression of transcription factors and signalling molecules, resulting in the formation of distinct developmental, and ultimately functional, domains.

**Results:**

We have discovered that the *Six3/6* family orthologue *Optix* is an essential regulator of neuroepithelial maintenance and patterning in the *Drosophila* brain. *Six3* and *Six6* are required for mammalian eye and forebrain development, and mutations in humans are associated with severe eye and brain malformation. In *Drosophila*, Optix is expressed in a sharply defined region of the larval optic lobe, and its expression is reciprocal to that of the transcription factor Vsx1. *Optix* gain- and loss-of-function affects neuroepithelial adhesion, integrity and polarity. We find restricted cell lineage boundaries that correspond to transcription factor expression domains.

**Conclusion:**

We propose that the optic lobe is compartmentalised by expression of Optix and Vsx1. Our findings provide insight into the spatial patterning of a complex region of the brain, and suggest an evolutionarily conserved principle of visual system development.

## Background

Maintaining the spatial organization of growing tissues represents a fundamental developmental challenge. Growth is essential for organogenesis, but must occur in a highly ordered fashion. One strategy is to organize stem cell or progenitor populations into epithelia, physically constraining the dividing cells [1]. For example, the stem cells that generate epidermal, intestinal and neural tissue form monolayered or pseudostratified epithelia that expand laterally to increase the pool of tissue specific precursor cells [2-4]. Growing epithelia are regionalised by zones of transcription factor and signalling molecule expression, which act in concert to confer specific fates and functions [5,6]. This is particularly evident during the development of the nervous system [7-11]. Proliferating tissues must be patterned as they grow, so that morphogenesis proceeds correctly and cells acquire the correct fate wherever they are located. However the precise molecular chain of events linking regionalisation, cell behaviour and tissue morphogenesis is only beginning to be uncovered.

The vertebrate central nervous system is patterned along its dorsal-ventral, anterior-posterior and medial-lateral axes [7-9]. For example, the vertebrate mid- and hindbrain are compartmentalised into lineage-restricted neuroepithelial domains called rhombomeres [12,13]. The formation of compartments ensures that cells in neighbouring rhombomeres segregate away from each other to maintain tissue boundaries. The embryonic neocortex is ‘arealized’, meaning that it is partitioned into regions with distinct functions, architectural organisation and gene expression signatures (reviewed in [14,15]). Each territory is subdivided by the action of signalling centres and transcription factor expression. This process of regionalisation is essential for the correct specification of neural precursors at different positions, and ultimately the formation of distinct neuronal subpopulations.

During the early development of the nervous system, proliferating neural progenitors are organised into a neuroepithelial sheet [4,16-18]. Neuroepithelial cells exhibit classical epithelial characteristics, such as apico-basal polarity and adherens junctions, and give rise to exclusively neural lineages. A comparison of the formation of the mammalian forebrain and the *Drosophila* optic lobe illustrates the striking evolutionary conservation of this mode of neural development [18-20]. In both systems, an early pool of symmetrically dividing neuroepithelial cells proliferates rapidly to expand neural progenitor numbers. Over time, there is a shift from neural stem cell expansion through symmetric division to asymmetric stem cell self-renewal, which promotes the maintenance of stem cell numbers and the production of differentiated neuronal and glial progeny.

The similarities between invertebrate and vertebrate neural development are not solely architectural. Notch signalling maintains neuroepithelial identity and regulates the balance between stem cell proliferation and differentiation in both the *Drosophila* optic lobe [21-27] and the mammalian cortex [28-30]. Thus fundamental organisational principles and molecular mechanisms are conserved between vertebrate and invertebrate neural development [19].

Despite the evolutionary conservation of many aspects of neurogenesis [10,19,31], it was not clear whether a process of spatial regionalisation occurs during the formation of the *Drosophila* optic lobe. Although less complex than the cerebral cortex, the optic lobe still contains enormous cellular diversity. The adult fly brain comprises roughly 150,000 neurons, of which approximately 60,000 belong to the visual system [32]. These neurons form the neural circuitry that receives and processes visual information from photoreceptors in the eye. The numbers, spatial organization and types of neurons produced must be tightly controlled to ensure the formation of functional visual circuits and preserve retinotopy - the spatial mapping of visual information from the retina to the brain [33].

Optic lobe neurons are formed during larval development by two proliferative neuroepithelia known as the inner and outer proliferation centres (IPC and OPC) [34,35]. Here we describe a new role for the homeobox gene *Optix* in regulating the spatial organisation of the OPC*. Optix* encodes a Six class homeodomain transcription factor with two vertebrate orthologues, *Six3* and *Six6*[36-38]. Thus far, *Optix* function has primarily been characterised in the context of *Drosophila* eye development, as it is a member of the gene regulatory network that coordinates proliferation and differentiation in the developing retina (the Retinal Determination Network; see reviews in [39-42]). *Optix* misexpression is sufficient to induce ectopic eye formation [43,44], and a recent study has shown that it is required for the progression of the morphogenetic furrow across the developing eye [45]. Work on *Optix* function during embryogenesis has also demonstrated that it has an important role in head specification and the regionalisation of the embryo [46].

We found that *Optix* has a striking expression pattern in the larval brain. Optix protein is expressed throughout larval development in a sharply defined segment of the optic lobe neuroepithelium. We observe that the OPC is pre-patterned by transcription factors, and that the sharp boundaries of Optix expression persist over the course of normal growth and Fat-Hippo-mediated overproliferation. Both gain and loss of *Optix* function induces cell sorting, the disruption of neuroepithelial tissue structure, and the formation of ectopic neuroepithelial rosettes. Furthermore, we find evidence of straight optic lobe lineage boundaries, which are defined by mutually exclusive transcription factor expression. These data have led us to propose a model in which *Optix* compartmentalises the brain and regulates neuroepithelial maintenance, polarity and survival in the optic lobe.

## Results

### The Six family homeodomain transcription factor Optix is expressed in the optic lobe neuroepithelium

**Figure 1:**
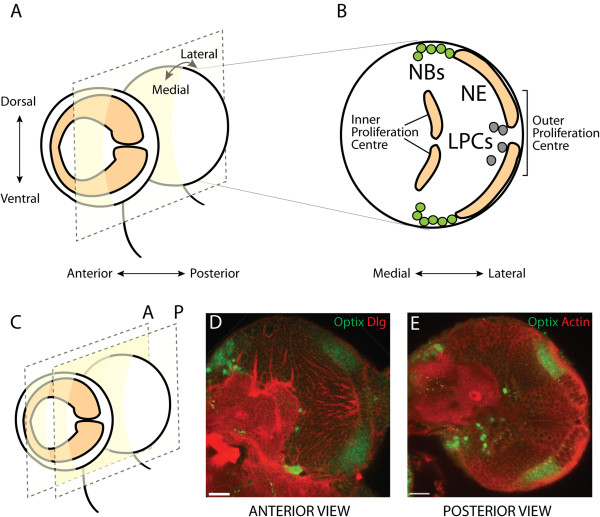
**Optix is expressed in half of the optic lobe neuroepithelium. (A)** Cartoon of a lateral view of larval third instar brain lobes. The optic lobe outer proliferation centre (OPC) is a horseshoe-shaped neuroepithelium (orange), which covers the lateral side of each brain lobe. A frontal cross-section is indicated by the dotted square. Dorsal-ventral, anterior-posterior and medial-lateral axes indicated by arrows. **(B)** Cartoon of a posterior frontal cross-section through a brain lobe at mid third instar. The OPC neuroepithelium (NE) generates two kinds of neural precursor: asymmetrically dividing medulla neuroblasts (NBs, green) and lamina precursor cells (LPCs, grey). Incoming retinal axons in the optic nerve enter through the central gap in the neuroepithelium. Medial-lateral axis indicated by arrows. **(C)** Cartoon of a lateral view from **(A)** showing the planes of two frontal cross-sections (dotted squares), one anterior (A) and one posterior (P). **(D)** Anterior confocal cross-section through a brain lobe at mid third instar. Cells are outlined in red by Discs large (Dlg) staining. Optix protein (green) is expressed across the neuroepithelium with a central gap. **(E)** Posterior confocal cross-section through a brain lobe at mid third instar. Cells are outlined in red by Actin staining (Phalloidin). Optix protein (green) is symmetrically expressed across the neuroepithelium with a sharp boundary.

We investigated which genes were expressed in the optic lobe neuroepithelium using transcriptome analysis [21,47]. This led to the identification of *Optix*, which was significantly upregulated in neuroepithelial cells compared to optic lobe neuroblasts. We analysed Optix protein expression in the optic lobe and found that it is strikingly enriched in the optic lobe outer proliferation centre. The outer proliferation centre (OPC) is a horseshoe-shaped neuroepithelium, which covers the lateral side of each brain lobe (Figure 1A, 1B). Each neuroepithelial arm gives rise to medulla neuroblasts (NBs) at the medial edge and lamina precursor cells (LPCs) at the lateral edge (Figure 1B). Optix is expressed in a symmetric domain in the two halves of the neuroepithelium (Figure 1C-E). It has a central gap in the anterior-most neuroepithelium (Figure 1D) and a particularly sharp posterior boundary of expression (Figure 1E). Interestingly, this posterior expression boundary abuts the Wingless signalling domain at the tips of the posterior OPC arms (Additional file 1; [48]). Optix protein expression is downregulated at the transition zone, where medial neuroepithelial cells become medulla neuroblasts and start dividing asymmetrically (Additional file 1). We noted Optix expression in other cell types in the brain, including glia and central brain neuroblast lineages (Additional file 2; [49]). These results indicated that Optix could potentially regulate brain development, in addition to its previously characterised role in retinal determination.

### Optix expression is maintained during neuroepithelial developmental expansion

**Figure 2:**
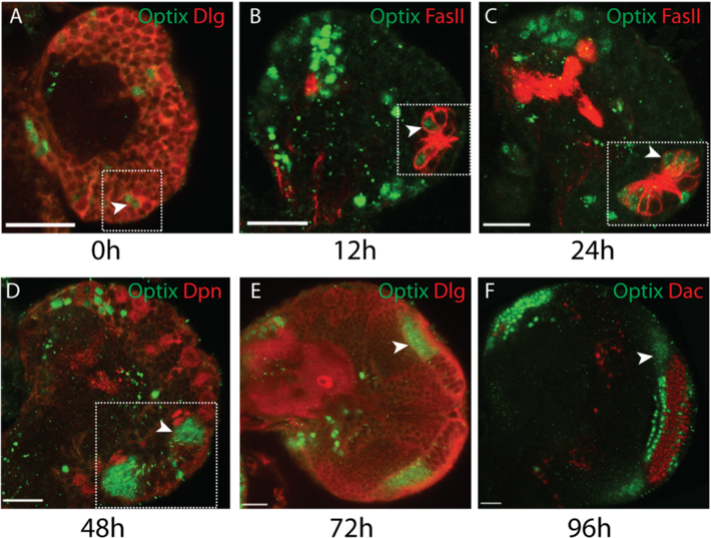
**Optix expression is maintained throughout optic lobe development. (A-F)** We detected Optix protein in the optic lobe from 0 to 96 hours after larval hatching (ALH). Optix expression was detected in the medial neuroepithelium with a sharp boundary of expression throughout neuroepithelial expansion (white arrowhead). Posterior cross-sectional views of the optic lobe are presented throughout. **(A-D)** The early optic lobe is outlined by dotted white box. **(A-C, D)** Cells are outlined by Discs large (Dlg) or Fasciclin II (Fas II) staining. **(D)** Deadpan (Dpn) is expressed in all neuroblasts. **(F)** Dachshund (Dac) is expressed in lamina precursor cells and lamina neurons. Scale bars: 20 μm.

In order to determine when Optix expression is established, we analysed Optix expression at different time points during larval development (Figure 2). The optic lobe arises from a small placode of cells in the embryo [35,50,51]. During embryogenesis these cells are quiescent, and they begin to proliferate just after larval hatching. The region expands and separates into two neuroepithelia, the outer and inner proliferation centres (OPC and IPC) [34,52]. We detect Optix expression in the optic lobe just after larval hatching (Figure 2A). Its expression persists in the same domain (roughly half of the neuroepithelium) as the neuroepithelium expands throughout larval development (Figure 2A-F). Its posterior expression boundary (Figure 1E) remains sharp during neuroepithelial expansion and differentiation. These results demonstrate that Optix expression is established and maintained from the beginning of larval development.

### Optix expression defines a neuroepithelial compartment

Developing tissues are frequently subdivided into compartments - groups of cells with a distinct identity, that do not intermix, and which may be lineage-restricted [6,53-59]. Compartment formation ensures that cells specified to different fates sort away from each other. The sharply defined zone of Optix expression in the neuroepithelium, and its early establishment, led us to hypothesise that the neuroepithelium is compartmentalised. In order to test this, we carried out lineage tracing in the neuroepithelium using *OptixGAL4* (*NP2631GAL4*[60]), which recapitulates the Optix expression pattern in the neuroepithelium (Additional file 3), and G-TRACE (‘GAL4 Technique for Real-time And Clonal Expression’; [61]). G-TRACE enables both historical and current *GAL4* expression to be visualized. It induces stable, heritably maintained EGFP expression in cells, allowing cell lineages to be mapped, while RFP expression labels cells currently expressing the GAL4 line.

**Figure 3:**
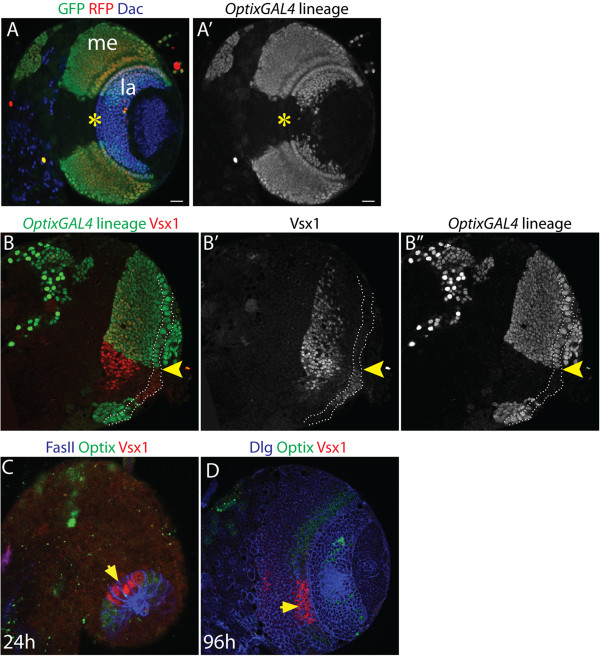
**Optix expression defines a restricted neuroepithelial lineage. (A)** Anterior view of a late third instar brain lobe. G-TRACE lineage tracing was performed using *OptixGAL4*. The GFP and RFP-labelled *OptixGAL4* lineage forms much of the medulla and part of the lamina neuronal cortices, aside from a clear central gap (yellow star). La, lamina cortex; me, medulla cortex. Lamina neurons are labelled by Dac (blue). **(B)** Anterior view of a late third instar brain lobe. The outer proliferation centre (OPC) neuroepithelium is outlined by the dashed white line. There is a sharp expression boundary (yellow arrowhead) between the *OptixGAL4* lineage derived from Optix-expressing neuroepithelial cells (GFP, green) and Vsx1-expressing neuroepithelial cells (red). (B’) Vsx1 is also expressed in medulla neurons that migrate and intermingle with medulla neurons derived from the *OptixGAL4* lineage. **(C)** Larval brain at 12 hours after larval hatching (ALH). Optix (green) and Vsx1 (red) are expressed in complementary domains. Fas II (blue) labels early neuroepithelial cells. Yellow arrow indicates Vsx1 domain. **(D)** Anterior view of larval brain at 96 hours ALH. Optix (green) and Vsx1 (red) are expressed in complementary domains. Cells outlined by Dlg (blue). Yellow arrow indicates Vsx1 domain.

The medulla and lamina are two of the visual processing ganglia in the adult optic lobe. Medulla and lamina neurons both derive from OPC neuroepithelial cells [34]. Lineage tracing analysis revealed that Optix-expressing neuroepithelial cells give rise to a neural lineage that forms much of the medulla cortex and also part of the lamina (Figure 3A-A’). The boundaries of the cell lineages derived from Optix-positive neuroepithelial cells are straight, with a clear central gap (Figure 3A-A’ , Figure 3B-B’). This gap corresponds to the anterior boundaries of Optix expression (Figure 1D). Interestingly it had previously been reported that the transcription factor Vsx1 is expressed in the central neuroepithelium [62]. Vsx1 is a *Drosophila* homologue of the homeodomain protein Chx10, which is essential for retinal progenitor cell proliferation and neuronal specification in vertebrates [63]. Vsx1 is expressed in a central population of OPC neuroepithelial cells and a subset of medulla neurons, and is required for neuroepithelial proliferation [62]. We found that Optix and Vsx1 are expressed in complementary neuroepithelial domains throughout optic lobe development (Figure 3B-D”). Vsx1 protein was also observed in medulla neurons, which migrate and mix with neurons derived from the *OptixGAL4* lineage. These lineage tracing results, and the complementary expression of Optix and Vsx1, support a model in which the optic lobe neuroepithelium is compartmentalised by transcription factor expression.

### Optix expression boundaries persist in conditions of tumorous growth

The posterior boundary of Optix expression in the neuroepithelium is maintained during the course of normal developmental expansion (Figure 2). Tumour growth can lead to disorderly tissue organisation and the disruption of regional boundaries [64,65], and so we hypothesised that neuroepithelial overgrowth might perturb the Optix expression pattern. We assessed this using a *dachsous* mutant allele (*ds*^*05142*^) to disrupt Fat-Hippo signalling (Figure 3). The Fat-Hippo pathway has an essential tumour suppressive role in regulating tissue growth and polarity [66-68], and is known to regulate proliferation and differentiation in the optic lobe neuroepithelium [27,69]. *dachsous* encodes a large atypical Cadherin which activates the Fat-Hippo signalling cascade through its receptor, Fat [70-73].

**Figure 4:**
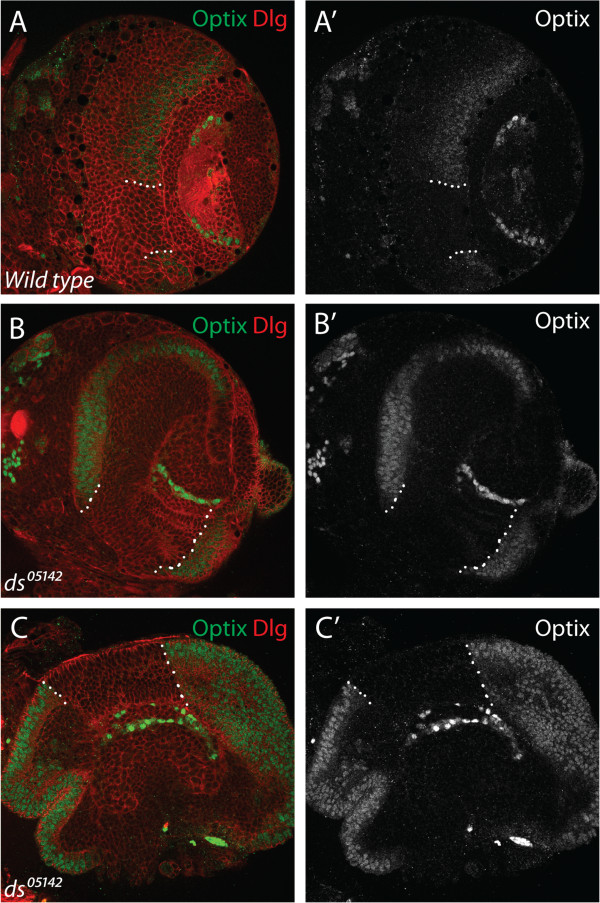
**Optix neuroepithelial expression boundaries are maintained during overproliferation. (A)** Anterior view of wild type third instar brain lobe shows Optix is expressed in the neuroepithelium with a central gap (indicated by dashed white lines). **(B, C)** Fat-Hippo signalling disruption in *ds*^*05142*^ mutants induces neuroepithelial overproliferation. Optix expression is still absent from the central domain of the anterior outer proliferation centre (OPC) neuroepithelium (dashed lines), and the boundary between Optix-positive and Optix-negative cells is straight. **(A-C)** Optix protein is in green, cells are outlined by Dlg staining in red.

*ds*^*05142*^ brains are grossly distorted owing to neuroepithelial overproliferation (Figure 4). Despite this, a sharp Optix expression domain is still visible in the optic lobe. This region is larger than in wild type as a consequence of tissue overgrowth, but it still possesses a discrete expression boundary (Figure 4A’ , B’ , C’). The persistence of the anterior expression boundary (which is usually ‘filled in’ by Vsx1 expression) suggests a mechanism is in place to ensure that Optix-expressing cells do not intermingle with their Optix-negative neighbours, even under conditions of extensive overgrowth. This supports our hypothesis that the neuroepithelium is subdivided into compartments defined by transcription factor expression, which are, remarkably, maintained during neoplasia.

### Optix represses Vsx1 in the neuroepithelium

**Figure 5:**
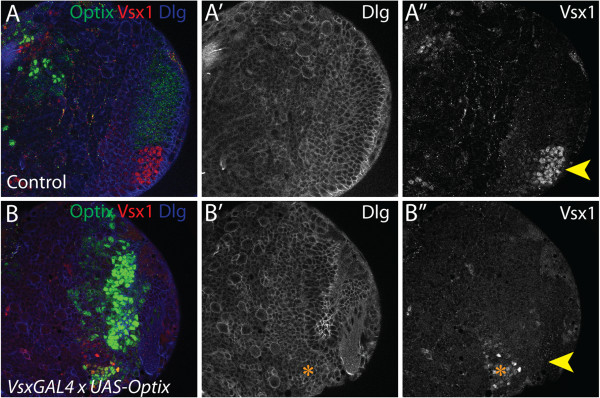
**Optix represses Vsx1 expression. (A-B”)** Anterior views of late third instar larval brains. Optix misexpression in the *VsxGAL4* neuroepithelial domain represses Vsx1 expression. Cells outlined by Dlg (blue), Optix protein in green, Vsx1 in red. **(B”)** Vsx1 expression is observed in neurons (orange star) but not in neuroepithelial precursors (yellow arrowhead).

Optix and Vsx1 proteins are expressed in complementary domains throughout optic lobe development, suggesting that they may be mutually exclusive (Figure 3C-D). Therefore, we tested the consequences of *Optix* misexpression on the Vsx1 domain (Figure 5A-B”). *Optix* was ectopically expressed in central Vsx1-expressing neuroepithelial cells using *VsxGAL4*[62]. This had two clear effects. Firstly, Vsx1 expression was lost from its central neuroepithelial domain (Figure 5B”). The only Vsx1-expressing cells observed in the optic lobe were not neuroepithelial, conforming instead to the wedge-shaped distribution of Vsx1-positive medulla neurons (orange star in Figure 5B”; [62]). Secondly, the morphology of the optic lobe neuroepithelium was distorted compared to wild type brains (Figure 5B’). These results show that ectopic *Optix* expression in the central neuroepithelial domain disrupts optic lobe patterning. Furthermore, they demonstrate that Optix is sufficient to repress Vsx1 expression, implying potential negative cross-regulation between Optix and Vsx1. This interaction could contribute to the sharp boundaries of Optix and Vsx1 expression in the anterior OPC neuroepithelium. However we did not observe ectopic Vsx1-positive cells in the Optix neuroepithelial compartment upon loss of *Optix* expression, either in RNAi knockdown conditions or null mutant clones (data not shown). This suggests that loss of *Optix* is not sufficient to induce Vsx1 expression within the Optix domain.

### *Optix* is necessary for neuroepithelial maintenance and cell survival

**Figure 6:**
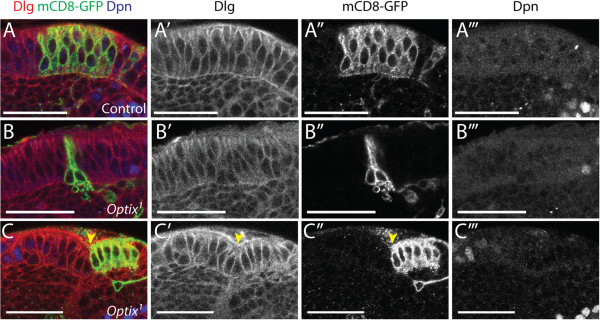
***Optix *is required for cell survival and adhesion within the neuroepithelium. (A-C”’)** Control (*FRTG13*) and *Optix*^*1*^ null mutant MARCM clones were induced in the neuroepithelium (labelled with mCD8-GFP in green). Cells are outlined by Dlg (red), neuroblasts labelled by Dpn (blue). **(A-A”’)** Wild type clones remain in the neuroepithelium. **(B-B”’)***Optix*^*1*^ mutant clones delaminate from the neuroepithelium and show signs of apoptosis, such as cellular fragmentation. **(C-C”’)***Optix*^*1*^ mutant clones induced in the lateral neuroepithelium (lateral to the lamina furrow, indicated by yellow arrowhead) do not delaminate or show signs of cell death. Posterior cross-sections are shown in all images. The apico-basal axis of the neuroepithelium is oriented vertically in each image, with the apical surface at the top and the basal surface at the bottom. Scale bars: 30 μm.

We analysed the effects of losing *Optix* function on optic lobe development. *Optix* null mutants are homozygous lethal and die during late embryogenesis [45]. We induced *Optix*^*1*^ null mutant MARCM (Mosaic Analysis with a Repressible Cell Marker) clones within the neuroepithelium (Additional file 4; [45,74]). Few mutant clones were recovered in the Optix-expressing region of the neuroepithelium. The *Optix* mutant clones that could be identified underwent basal extrusion from the neuroepithelium, indicated by their basal cell bodies and elongated apical stalks (Figure 6B, B”). In contrast, control clones or *Optix* mutant clones induced where *Optix* is not expressed appeared wild type (Figure 6A-A”’, 6C-C”’). *Optix* mutant clones showed signs of cell death, including fragmentation, which were not evident in control clones (Figure 6B-B”) They were positively labelled by TUNEL (Terminal deoxynucleotidyl transferase dUTP Nick End Labelling) (Additional file 4) and so we concluded that *Optix* is required for cell survival in a distinct region of the optic lobe.

**Figure 7:**
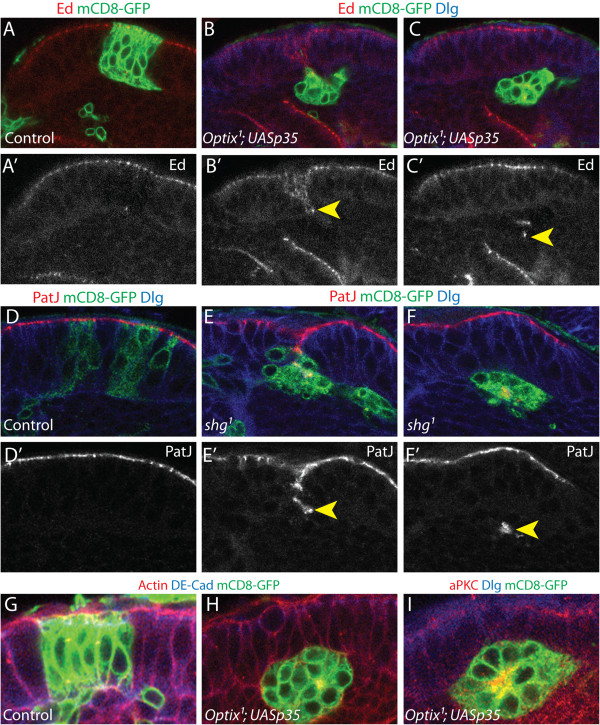
**Loss of adhesion leads to ectopic neuroepithelial rosette formation. (A-C’)** Cell death in *Optix*^*1*^ null mutant MARCM clones was rescued by expression of *p35*, which inhibits apoptosis. Clones are labelled with mCD8-GFP (green), Echinoid labels adherens junctions (Ed, red), cells are outlined by Dlg (blue). **(A-A’)** Control clones expressing *p35* remain in the neuroepithelium. **(B-C’)***Optix*^*1*^ mutant clones expressing *p35* form ectopic neuroepithelial rosettes in the underlying medulla cortex. **(D-F)** Null mutant clones for DE-Cadherin (*shg*^*1*^) delaminate basally from the neuroepithelium to form rosettes below in the underlying medulla cortex **(E, F)**, in contrast to *FRTG13* control clones. **(D)** Clones are labelled with mCD8-GFP (green), PatJ labels adherens junctions (PatJ, red), cells outlined by Dlg (blue). **(G-I)** Apically localised proteins cluster at the centre of the neuroepithelial rosettes formed by rescued *Optix*^*1*^; *UASp35* clones. **(G-H)** Clones are labelled with mCD8-GFP (green), DE-Cadherin labels adherens junctions (DE-Cad, blue), cells outlined by Phalloidin (F-Actin, red). **(I)** Clone is labelled with mCD8-GFP (green), cells outlined by Dlg (blue), aPKC is an apically localised protein (aPKC, red). **(A-I)** Posterior cross-sections are shown in all images. The apico-basal axis of the neuroepithelium is oriented vertically in each image, with the apical surface at the top and the basal surface at the bottom.

It was not clear whether the cell death detected in *Optix* mutant clones was a result of basal extrusion from the neuroepithelium, or whether these cells were extruded because they were undergoing apoptosis. In order to ascertain this, we rescued *Optix* mutant clones from cell death with baculovirus P35. P35 impairs apical caspase function and inhibits the pro-apoptotic enzyme cascade [75]. Rescuing cell death did not prevent the basal extrusion of *Optix* mutant clones. Instead, the rescued *Optix*^*1*^*; UASp35* mutant cells sort away from the surrounding wild type neuroepithelium (Figure 7B-C) and form ectopic neuroepithelial rosettes in the underlying differentiated cell layer of the medulla cortex (Figure 7C, 7H, 7I). Apically localised proteins and components of adherens junctions, such as Echinoid [76] and DE-Cadherin [77-81], are clustered in the centre of these rosettes (yellow arrowheads, Figure 7B-C). In contrast, control clones expressing *p35* remain within the neuroepithelium and do not form rosettes (Figure 7A,G). These results indicated that extrusion of *Optix* mutant cells from the neuroepithelium was not a consequence of cell death, as it occurred even when apoptosis was blocked. We therefore concluded that *Optix* is selectively required for the maintenance of neuroepithelial adhesion and cell survival, within its own domain of expression. This reinforces the notion that *Optix* expression serves to define a neuroepithelial compartment.

### E-Cadherin-mediated adhesion is required for neuroepithelial maintenance

The extrusion of *Optix* mutant clones from the neuroepithelium, and their subsequent rosette formation, suggested that *Optix* might regulate neuroepithelial adhesion. We investigated the effects of disrupting cell adhesion in the neuroepithelium by analysing loss of *Drosophila E-Cadherin* (*DE-Cadherin*) function. DE-Cadherin is a crucial component of cellular adherens junctions, and is necessary for maintaining adhesion in a variety of epithelial tissues [77,80-82]. We induced neuroepithelial clones that are mutant for *shotgun* (*shg*), which encodes DE-Cadherin [79]. Interestingly, we observed similar cellular responses to loss of both *Optix* and *shg* function (compare Figure 7B-C to 7E-F). *shg*^*1*^ mutant clones were basally extruded from the neuroepithelium and formed rosettes beneath the medulla cortex (Figure 7E, 7F). There were also signs of fragmentation and cell death (Figure 7F). This supported the idea that neuroepithelial cells sort away from each other when adhesion is altered, and that *Optix* regulates optic lobe compartmentalisation by maintaining neuroepithelial adhesion.

Many studies have reported that *Notch* mutant clones delaminate from the neuroepithelium and transform into ectopic medulla neuroblasts [21-27]. When neuroepithelial cells become neuroblasts, they reorient their spindle poles and dismantle their adherens junctions [52]. *Notch* mutant clone delamination is likely a consequence of a loss in adhesion as neuroepithelial cells transform into neuroblasts. However, Wang *et al*. have reported that a loss of epithelial integrity is not always associated with premature neuroblast formation [24]. Neither *Optix*^*1*^ nor *shg*^*1*^ mutant cells showed any signs of differentiation or neurogenesis. In particular, they did not switch on expression of the neuroblast-specific transcription factor Deadpan (Figure 6B”’). This led us to conclude that inducing cells to leave the neuroepithelial niche by modulating adhesion is not sufficient to induce the transition to a neuroblast fate, consistent with Wang *et al*’s study of Notch function [24]. The clustering of *Optix* and *shg* mutant neuroepithelial cells into rosettes suggests that these clones have partially lost adhesion but are still able to adhere to each other. Perhaps this retention of adhesion contributes to the maintenance of neuroepithelial character.

### *Optix* misexpression disrupts neuroepithelial architecture

**Figure 8:**
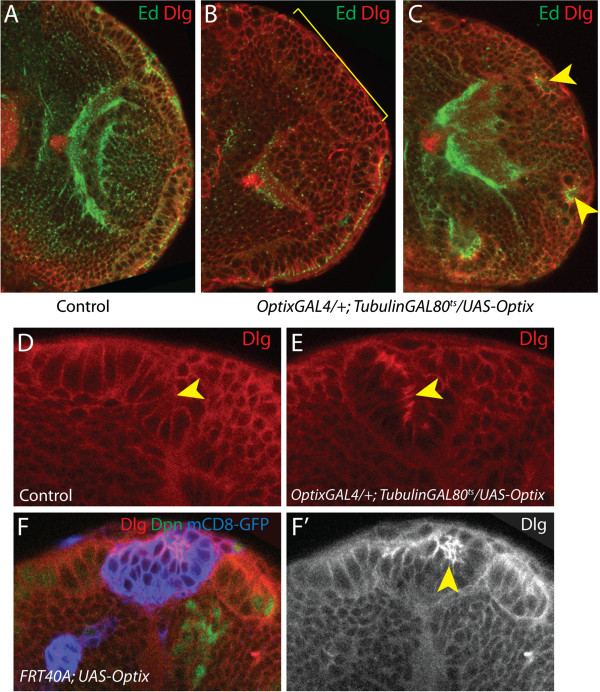
***Optix *misexpression disrupts neuroepithelial organisation. (A-C)** Posterior cross-sections of late third instar brain lobes. Cells are outlined by Dlg (red) and Ed staining labels apical cell surfaces (green), indicating the apico-basal axis of the tissue with respect to the brain. *Optix* misexpression using *OptixGAL4* severely disrupts neuroepithelial architecture. Multilayering of neuroepithelial cells **(B)** and rosette formation (**C**, yellow arrowheads) were observed, in contrast to the regular pseudostratified structure of wild type outer proliferation centre (OPC) neuroepithelium **(A)**. **(D, E)***Optix* misexpression alters Dlg (red) levels and localisation. Higher levels of apical Dlg are observed when *Optix* is misexpressed (yellow arrowhead in **E**). **(F, F’)***Optix* misexpression clones were induced in the neuroepithelium. These clones form rosettes in which Dlg accumulates apically (yellow arrowhead). Clones labelled with mCD8-GFP (blue), cells stained for Dlg (red) and Dpn (green). **(D-F)** The apico-basal axis of the neuroepithelium is oriented vertically in each image, with the apical surface at the top and the basal surface at the bottom.

Removing *Optix* from cells surrounded by wild type *Optix*-expressing neighbours results in basal extrusion and cell sorting. To explore the effects of increased *Optix* expression on neuroepithelial cell behaviour, we overexpressed *Optix* in the neuroepithelium. *Optix* was misexpressed using a temperature-sensitive driver combination either throughout the OPC neuroepithelium (with *c855aGAL4*) or in the neuroepithelial domain where it is usually expressed (with *OptixGAL4*). *Optix* misexpression using both drivers induced multi-layering of the OPC neuroepithelium (Figure 8B; Additional file 5), compared to its wild type pseudostratified architecture [52,83]. Neuroepithelial cells rounded up and lost their columnar epithelial shape. Apical polarity markers such as Echinoid were lost from these multilayered epithelia (Figure 8B). In some instances *Optix* misexpression caused the arms of the neuroepithelium to curve and round up on themselves. This resulted in the formation of rosette-like structures, with the apical centres of cells clustered together in the centre, as indicated by Echinoid localisation (Figure 8C). Thus *Optix* misexpression resulted in a loss of columnar neuroepithelial apico-basal polarity.

We tested the effects of *Optix* misexpression further by inducing overexpression clones and analysing their behaviour in the neuroepithelium. *Optix* misexpression clones formed neuroepithelial rosettes (Figure 8F-F’). Rosette formation upon clonal *Optix* misexpression was also observed in other epithelial tissues, including the imaginal eye and leg discs (Additional file 5C-D”). We noticed that the localisation of the PDZ domain protein Discs large (Dlg) was altered upon *Optix* misexpression (Figure 8F). Dlg outlines cell cortices in wild type OPC neuroepithelial cells [52]. In *Optix* misexpression-induced rosettes, high levels of Dlg were observed towards the centre of the rosette, accumulating at the apical surfaces of cells (Figure 8D-F’ , Additional file 5). This was also observed when *Optix* was misexpressed across the neuroepithelium (Figure 8E). Thus *Optix* misexpression is sufficient to disrupt Dlg localisation and the pseudostratification of the optic lobe neuroepithelium, and we concluded that *Optix* is a potent regulator of cell polarity and adhesion.

## Discussion

### The optic lobe is compartmentalised by transcription factor expression

**Figure 9:**
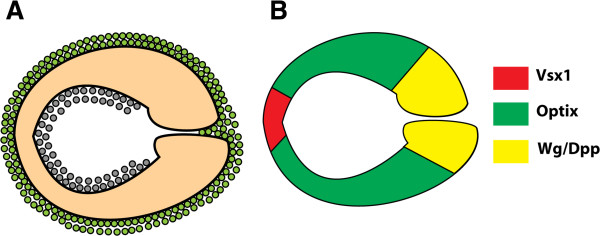
**Model for regional neuroepithelial compartments in the optic lobe. (A)** Lateral view of the outer proliferation centre (OPC) neuroepithelium (orange), which generates both medulla neuroblasts (green) and lamina precursor cells (grey). **(B)** The same lateral view of the neuroepithelium, colour coded by the regionalised expression of transcription factors and signalling pathways. Vsx1 (red) is expressed in a central neuroepithelial domain; Optix (green) is expressed in a symmetrical domain on either side. Wingless signalling is active at the tips of the OPC, and activates the Dpp pathway in turn [48].

The sharply delineated boundaries of Optix expression in the neuroepithelium, and of the lineages derived from this region, suggest that the OPC is compartmentalised. Compartments are classically defined as lineage-restricted populations of cells that do not mix and which may be specified by the expression of a transcription factor or selector gene (reviewed in [53-55,57,58]). We propose that neural stem cells in the OPC have distinct regional identities conferred by the expression of specific transcription factors (Figure 9). This is supported by the reciprocal expression patterns of Optix and Vsx1, and the fact that that *Optix* misexpression represses Vsx1 expression, raising the possibility that these transcription factors act as selector genes for distinct neuroepithelial regions. Early *Optix* and *Vsx1* expression in the optic lobe could specify ‘founder populations’ of neuroepithelial cells (Figure 3C), which expand through rounds of symmetric division and ultimately establish the proportions of different OPC regions. The posterior Optix expression boundary is reciprocal to the Wingless signalling domain at the tip of the neuroepithelium. The highly regionalised signalling activity of the Wingless and Dpp pathways also contributes to OPC patterning (Figure 9; [48]).

The cellular mechanisms that control cell sorting and maintain compartment boundaries are not fully understood. Several hypotheses have been proposed, including differential adhesion, differential rates of proliferation, and physical barriers between compartments formed by localised actomyosin cables [58]. In the embryonic mouse forebrain, Cadherin6 and R-Cadherin play an important role in maintaining the compartment boundary between progenitors that form the cerebral cortex and the striatum [84]. Loss of either *Optix* or *DE-Cadherin* function induces basal extrusion from the neuroepithelium and the formation of neuroepithelial rosettes in the medulla cortex. This phenotypic similarity suggests that *Optix* regulates intercellular adhesion. A number of Cadherins and other cell adhesion molecules have enriched expression in the OPC neuroepithelium [21,47,85]. However, no adhesion molecules have been identified that are regulated by Optix, nor that are expressed in a similarly restricted pattern across the OPC. It is possible that Optix regulates the transcription or post-transcriptional modification of cell adhesion molecules indirectly through other target genes.

Loss and gain of *Optix* function lead to altered neuroepithelial polarity and, in some cases, the formation of rosettes, suggesting that *Optix* expression levels must be tightly regulated. Observations of cell sorting upon changes in *Optix* expression provide further evidence of tissue compartmentalisation in the optic lobe. This behaviour is reminiscent of the cell sorting phenotypes seen when cells are incorrectly specified within a tissue compartment, for example in the *Drosophila* wing imaginal disc. The wing disc is divided into anterior and posterior compartments, and expression of the transcription factor Engrailed confers posterior compartment identity [86-89]. *Engrailed* mutant clones induced in the posterior compartment sort away from their neighbours, moving across the compartment boundary into the anterior domain. *Optix*-expressing cells did not mix with *Optix*-negative cells even in conditions of hyperplastic overgrowth in *dachsous* mutant brains, suggesting that mechanisms are in place to prevent the intermingling of these neuroepithelial populations.

### Spatial and temporal patterning of the neuroepithelium regulates visual system formation

Retinotopy - the mapping of visual inputs from the retina to the brain in order to preserve spatial information - is the essential overarching principal of visual system development and organisation. The adult optic lobe contains an estimated 60,000 neurons [32], of which there are at least 70 different subtypes [90,91]. A system of spatio-temporal patterning must be at work in neural progenitors to ensure the production of correctly specified neurons at the right time and place. Indeed two groups recently identified a temporal cascade that patterns neural fates in the optic lobe [92,93]. Optic lobe medulla neuroblasts express a sequence of transcription factors as they age. This clock mechanism specifies neuronal fates in a birth order-dependent manner, in a similar manner to the temporal cascade at work in embryonic neuroblasts [94-106]. Although the transcription factor code itself is different, the same biological mechanism is employed to generate cell fate diversity in both systems (reviewed in [107]).

The OPC neuroepithelium is regionalised by the expression of the transcription factors Optix and Vsx1, in addition to Wingless and Dpp activity (Figure 9). These regional inputs may specify medulla neuroblast fates, and could be combined with the temporal transcription factor cascade to generate diverse neuronal subtypes in the medulla. It is evident that the OPC is regionalised from early larval development (Figure 2, Figure 3C). Signalling centres in the larval neuroepithelium could induce or maintain transcription factor expression, and hence establish compartment boundaries. Indeed there are many candidate pathways which are active in the OPC, including Dpp, Wingless, Fat-Hippo, Notch, EGFR and JAK/STAT [21-27,69,108-110]. It remains to be determined how these different inputs are integrated to regulate neural stem cell fates in the optic lobe.

### *Optix/Six3/6* has a conserved role in neural development

Neural stem cells are organised into neuroepithelia in most developing nervous systems. These tissues constrain neural precursors architecturally, influencing how they divide and providing a field of cells for molecular patterning. Our findings demonstrate a critical role for *Optix* in regulating neuroepithelial organisation, adhesion and patterning during optic lobe development. We have shown that *Optix* is essential for cell survival and adhesion in the OPC neuroepithelium, and that ectopic *Optix* expression perturbs neuroepithelial organisation. These results further our understanding of how *Optix* regulates visual system development in the *Drosophila* brain as well as the eye, and they indicate that early neuroepithelial compartmentalisation is a crucial strategy for the development of the optic lobe.

Our findings have direct implications for vertebrate neural development. *Optix* and its orthologues, *Six3* and *Six6*, encode Six family homeodomain transcription factors that have highly conserved roles in neuroepithelial patterning and regionalisation. *Six3* serves as a marker of anterior regional identity across the three major bilaterian clades [111-121]. *Six3* and *Six6* are crucial for vertebrate eye and forebrain development (reviewed in [122]). *Six6* knockout mice exhibit retinal and pituitary hypoplasia [123] and *Six3* mutant mice lack most of the forebrain [124]. Further, *Six3/6* mutations in humans are associated with a number of severe eye and brain defects including micropthalmia (small eyes), anopthalmia (absence of one or both eyes), pituitary defects and holoprosencephaly (failure of the forebrain to separate into two hemispheres) [125-135].

The important roles *Six3* and *Six6* play in mammalian retinal and forebrain development, together with evidence of compartmentalisation in the vertebrate brain [84], suggest that the mechanisms underlying visual system development and neuroepithelial patterning are highly conserved from invertebrates to vertebrates. Further investigation into how Optix controls neuroepithelial development may enhance our understanding of *Six3/6*-associated congenital brain malformation, providing a genetically tractable model for studying visual system development at the cellular and molecular level.

## Conclusions

Here we show that neuroepithelial progenitors of the *Drosophila* optic lobe OPC are spatially patterned by the expression of two highly conserved transcription factors: Vsx1 and Optix. *Optix* defines a neuroepithelial compartment, with a territory of expression that persists throughout physiological growth and Fat-Hippo pathway-induced overproliferation. Like other developing epithelia, such as the wing imaginal disc, this region of the brain uses compartmentalisation as a mechanism for tissue organisation. *Optix* is required for neuroepithelial cell survival and adhesion, and its loss results in cell sorting and ectopic neuroepithelial rosettes. *Optix* misexpression is sufficient to repress Vsx1 expression and induce epithelial multilayering, disrupting neuroepithelial architecture. The Optix orthologues Six3 and Six6 play a critical role in vertebrate eye and brain development, suggesting that the spatial patterning of the visual system may be conserved from invertebrates to vertebrates.

## Methods

### Fly lines and staging

Flies were raised on standard cornmeal medium at 25°C or at room temperature (within a range of 21 to 25°C). OregonRS flies were used as wild type controls, unless stated otherwise. Temperature shift experiments were carried out by shifting flies from a permissive temperature of 18°C to a non-permissive temperature of 29°C. Larvae were reared at the required temperature to the desired stage, based on size and time after hatching: just hatched or early first instar (24 to 28 hours after egg laying), first instar (0 to 23 hours after larval hatching, ALH), second instar (23 to 46 hours ALH), mid third instar (69 to 75 ALH) or late third instar (93 to 99 hours ALH).

The following transgenic fly lines were used: *w; c855aGAL4; tubGAL80*^*ts*^[52,136,137], *w; NP2631; tubGAL80*^*ts*^[60], *InscuteableGAL4 (GAL4*^*1407*^*)*[138], *yw; ; UAS-Optix* (gift from J Kumar; [44]), *w; Optix*^*1*^*/CyO* (gift from R Chen; [45]), *yw hs-FLP; FRT40A, tubGAL80*^*LL10*^*/CyO,ActGFPJMR1; tubGAL4*^*LL7*^*,UASmCD8::GFP*^*LL6*^*/TM6B*, *w; FRT40A/CyO; TM6B/MKRS*, *yw hs-FLP; FRTG13,tubGAL80/CyO,ActGFP; tubGAL4,UAS-mCD8::GFP/TM6B* (all gifts from B Bello), *w; FRT40A/CyO; UAS-Optix/TM6B*, *w; FRTG13/SM6a* (Bloomington 1958), *w; ;UAS-p35* (Bloomington 5073), *w; FRTG13/CyO; UAS-p35*, w; *FRTG13,Optix*^*1*^*; UAS-p35/TM6B*, *w; ;UAS-RFP,UAS-flp,Ubi-p63E FRT > STOP > FRT EGFP*[61] (Bloomington 28281), *w; FRTG13 shg*^*1*^*bw sp/CyO,ftz-lacZ*[139], *ds*^*05142*^*/CyO* (Bloomington 11394), *w,VsxGAL4; tubGAL80*^*ts*^*/CyO* (gift from C Desplan; [62]), *yw hs-FLP; sp/CyO; WingfulGAL4/TM6B*[140], *w; dppGAL4*[141].

### Genetic crosses

Mutant and misexpression clones of cells were induced using the MARCM system [74]. MARCM clones were induced at 12 to 24 hours ALH by heat shocking larvae on fly food plates. The following regime was used: 5 minutes at 37°C, 5 minutes at room temperature, 15 to 30 minutes at 37°C. The exact length of the heat shock depended on the MARCM clone induction line being used. Clones were analysed at 72 to 96 hours ALH. For *Optix* misexpression experiments, embryos of the genotype *w; tubGAL80*^*ts*^*/+; c855aGAL4/UAS-optix* and *w; OptixGAL4/tubGAL80*^*ts*^*; UAS-Optix/+*were collected at 18°C and shifted to 29°C between 24 and 48 hours ALH to induce overexpression. Brains were dissected between 72 and 96 hours ALH. For G-TRACE lineage tracing experiments, embryos of the genotype *w; OptixGAL4/+; UAS-RFP, UAS-flp, Ubi-p63E FRT > STOP > FRT EGFP* were collected and raised at 25°C, and brains were dissected at 96 hours ALH.

### Immunohistochemistry

Larval brains were dissected in PBS and fixed for 20 minutes at room temperature in 4% formaldehyde and fixation buffer (PBS, 5 mM MgCl_2_, 0.5 mM EGTA). After fixation, brains were rinsed and washed in 0.3% PBS-Triton-X100 (PBT). Samples were blocked in 10% normal goat serum (NGS) in 0.3% PBT at room temperature and incubated with the primary antibody overnight at 4°C. Brains were then washed in 0.3% PBT, blocked in 10% NGS/0.3% PBT and incubated with the secondary antibody overnight at 4°C. After incubation with the secondary antibody, tissues were washed in 0.3% PBT and cleared at 4°C in Vectashield (Vector Laboratories, Burlingame, CA, USA). Brains were mounted in Vectashield.

Primary antibodies used in this study were: mouse anti-Dlg (1:50), mouse anti-Fas II (1:20), mouse anti-Dachshund (1:100), mouse anti-Eyes absent (1:75), mouse anti-Repo (1:70) (all from the Developmental Studies Hybridoma Bank, University of Iowa), rabbit anti-Optix (1:500, gift from F Pignoni; [142]), guinea pig anti-Dpn (1:1,000; gift from J Skeath), chicken anti-GFP (1:2,000, Abcam, Cambridge, UK), guinea pig anti-dVsx1 (1:750, gift from H Lipshitz; [62]), rabbit anti-Echinoid (1:50; gift from A Jarman; [143]), rabbit anti-PatJ (1:500, gift from W Zhou; [144]), rabbit anti-nPKC (1:500, Santa Cruz Biotechnology, Dallas, TX, USA), rabbit anti-Eyeless (1:300, gift from U Walldorf), rabbit anti-Scribble (1:2,000; gift from C Doe; [145]), rabbit anti-Ph3 (1:100, Upstate Biotechnology, Lake Placid, NY, USA). Fluorescently conjugated secondary antibodies were used at a dilution of 1:200 (Alexa405, Alexa488, Alexa546, Alexa568, Alexa633; Molecular Probes, Eugene, OR, USA). F-actin was labeled using Alexa fluorophore-conjugated Phalloidin (Phalloidin-546, 1:300, Molecular Probes). TUNEL staining of larval brains was carried out using the ApopTag^®^ Red *In Situ* Apoptosis Detection Kit (EMD Millipore, Billerica, MA, USA) according to the manufacturer’s instructions.

Images were acquired with a Leica TCS SP2 or SP5 confocal microscope (Leica Microsystems, Wetzlar, Germany) and analysed with Imaris (Bitplane, Zurich, Switzerland) or Fiji [146]. Figures and illustrations were assembled using Adobe Photoshop CS3 and Adobe Illustrator CS3 (Adobe Systems, San Jose, CA, USA).

## Abbreviations

ALH: after larval hatching; G-TRACE: GAL4 technique for real-time and clonal expression; GFP: green fluorescent protein; IPC: inner proliferation centre; LPC: lamina precursor cells; MARCM: Mosaic Analysis with a Repressible Cell Marker; NB: neuroblast; NE: neuroepithelium; OPC: outer proliferation centre; RFP: red fluorescent protein; TUNEL: Terminal deoxynucleotidyl transferase dUTP Nick End Labelling; PBS: phosphate-buffered saline; PBT: PBS-Triton-X100; NGS: normal goat serum.

## Competing interests

The authors declare that they have no competing interests.

## Authors’ contributions

KSG and AHB conceived the study and designed the experiments. KSG conducted the expression analysis, lineage tracing, *Optix* gain and loss of function studies, and *shg* loss of function analysis. KSG and AHB wrote the manuscript. Both authors read and approved the final manuscript.

## Supplementary Material

Additional file 1 **Regionalised Optix expression in the optic lobe.** (A) Wingless signalling is active at the lateral edge of the neuroepithelium (between white arrowheads). Optix expression starts just medially to the edge of the Wingless signalling zone (yellow arrowhead). *WingfulGAL4* driving *UAS-mCD8GFP* is the Wingless reporter used [140]. Scale bars: 20 μm. (B) Dpp is expressed more medially than Wingless (white arrowheads), and Optix expression begins in the middle of the Dpp signalling zone (yellow arrowhead). (A, B) Scale bars: 40 μm. Posterior cross-sections through the optic lobe are shown. (C) Optix expression in the medial neuroepithelium is downregulated at the transition zone (white arrowhead), where neuroepithelial cells transform into medulla neuroblasts. Medulla neuroblasts are labelled by *Inscuteable GAL4* driving *UAS-mCD8GFP* (*Insc-GAL4*, red) and the neuroblast-specific transcription factor Deadpan (blue), and Optix protein is in green. Click here for file

Additional file 2 **Optix is expressed in glia and neuroblast lineages.** (A) Optix protein is expressed in optic lobe glial cells, including the epithelial and marginal glia (white arrows). Cells labelled with Phalloidin, which stains F-actin (red), the pan-glial transcription factor Repo (blue) and Optix protein (green). (B) Optix is expressed in central brain neuroblast lineages. It can be seen primarily in Type II neuroblasts (white arrows), and in the differentiating progeny of these cells. It was also visible in approximately 1 Type I neuroblast per brain lobe. Neuroblast lineages are labelled by *Inscuteable GAL4* driving *UAS-mCD8GFP* (green), the neuroblast-specific transcription factor Dpn (blue) is expressed in Type I and II neuroblasts as well as Type II lineage intermediate neural progenitors, and Optix is in red. Click here for file

Additional file 3 ***OptixGAL4* recapitulates Optix protein expression.** (A-A”) Posterior frontal cross-section of the optic lobe. *OptixGAL4* driving *UAS-mCD8GFP* (red) showed a similarly well-defined expression pattern in the neuroepithelium to Optix protein (green). Both protein and the *GAL4* line have sharp expression boundaries in the OPC neuroepithelium. Click here for file

Additional file 4 ***Optix* null mutant clones do not express Optix protein and undergo apoptosis.** Description: *Optix*^*1*^ mutant MARCM clones were induced in the neuroepithelium (labelled with mCD8-GFP in green, Dlg in blue). (A-A’) *Optix*^*1*^ clones do not stain for Optix protein (red), indicating that they are null mutant clones. (B-B”) *Optix*^*1*^ clones in the neuroepithelium undergo apoptosis. They are basally extruded from the neuroepithelium and stain positively for TUNEL (yellow arrow). Click here for file

Additional file 5 ***Optix* misexpression induces multilayering and clonal cell sorting.** (A-B’) *Optix* misexpression throughout the neuroepithelium (with *c855aGAL4*) induces multilayering. Neuroepithelial cells take on a more rounded appearance, as opposed to their wild type columnar morphology. Cells are outlined by Dlg (red), neuroblasts stained by Dpn (green) and mitotic cells labelled by phospho-histone-H3 (PH3, blue). (C-D”) *Optix* misexpression clones induced in imaginal eye (C) and leg (D) discs form epithelial rosettes. Cells are outlined by Discs large staining (Dlg, red), clones labelled with mCD8-GFP (GFP, green), Optix stained in blue. (C”) Upon misexpression, Optix protein levels are very high (yellow arrowhead) compared to endogenous levels (white arrowhead). Misexpression clones sort away from their neighbours, and apical constriction and increased apical accumulation of Dlg protein (white arrowhead in D’) is visible. Scale bars: 20 μm. Click here for file
