# Supplementary figures and images for: Optix defines a neuroepithelial compartment in the optic lobe of the Drosophila brain
Source: Neural Dev. 2014 Jul 29;9:18. doi: 10.1186/1749-8104-9-18 (PMC4127074; doi:10.1186/1749-8104-9-18)

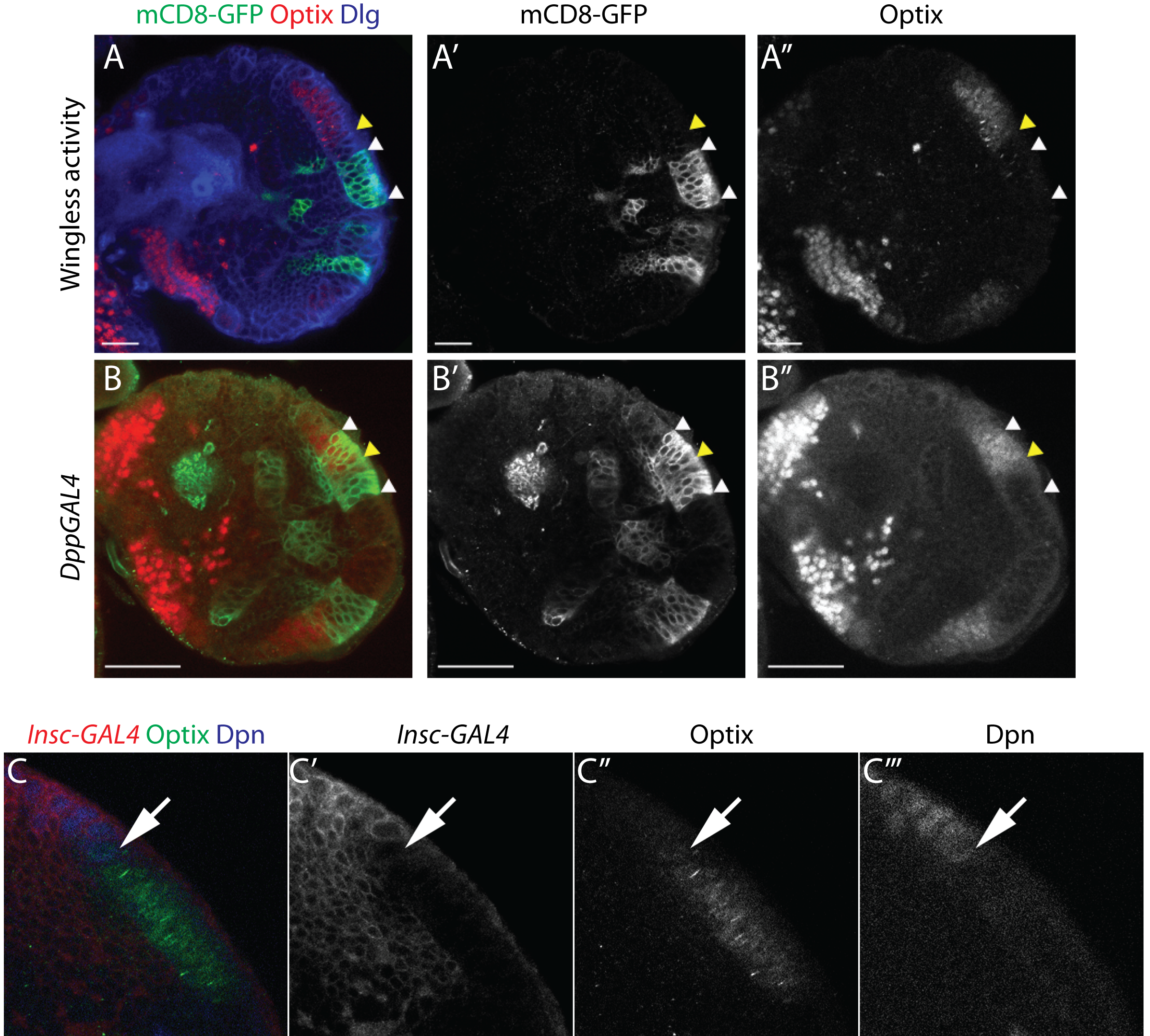

Supplement: Additional file 1 — Regionalised Optix expression in the optic lobe. (A) Wingless signalling is active at the lateral edge of the neuroepithelium (between white arrowheads). Optix expression starts just medially to the edge of the Wingless signalling zone (yellow arrowhead). WingfulGAL4 driving UAS-mCD8GFP is the Wingless reporter used [140]. Scale bars: 20 μm. (B) Dpp is expressed more medially than Wingless (white arrowheads), and Optix expression begins in the middle of the Dpp signalling zone (yellow arrowhead). (A, B) Scale bars: 40 μm. Posterior cross-sections through the optic lobe are shown. (C) Optix expression in the medial neuroepithelium is downregulated at the transition zone (white arrowhead), where neuroepithelial cells transform into medulla neuroblasts. Medulla neuroblasts are labelled by Inscuteable GAL4 driving UAS-mCD8GFP (Insc-GAL4, red) and the neuroblast-specific transcription factor Deadpan (blue), and Optix protein is in green. [file 1749-8104-9-18-S1.png]

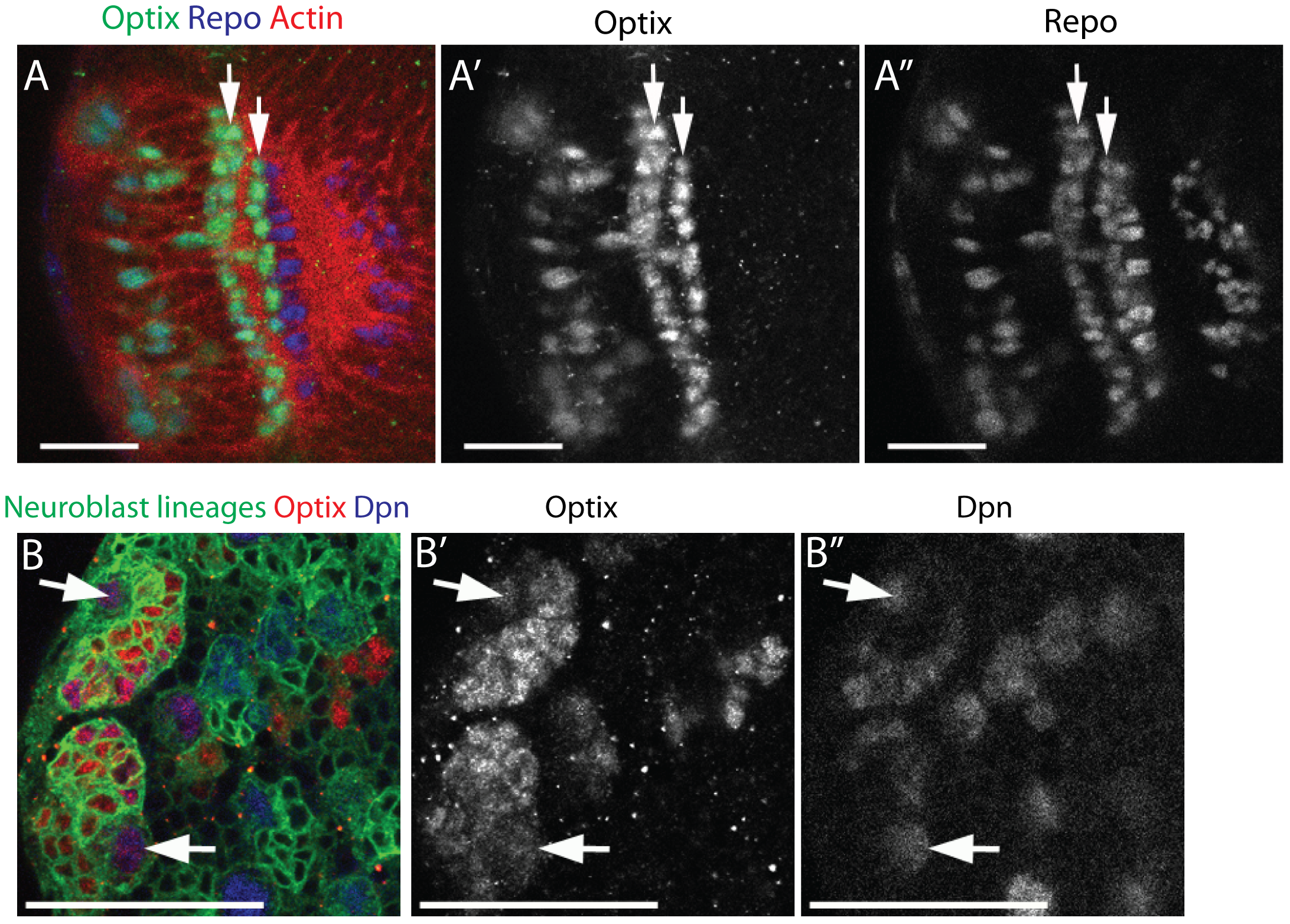

Supplement: Additional file 2 — Optix is expressed in glia and neuroblast lineages. (A) Optix protein is expressed in optic lobe glial cells, including the epithelial and marginal glia (white arrows). Cells labelled with Phalloidin, which stains F-actin (red), the pan-glial transcription factor Repo (blue) and Optix protein (green). (B) Optix is expressed in central brain neuroblast lineages. It can be seen primarily in Type II neuroblasts (white arrows), and in the differentiating progeny of these cells. It was also visible in approximately 1 Type I neuroblast per brain lobe. Neuroblast lineages are labelled by Inscuteable GAL4 driving UAS-mCD8GFP (green), the neuroblast-specific transcription factor Dpn (blue) is expressed in Type I and II neuroblasts as well as Type II lineage intermediate neural progenitors, and Optix is in red. [file 1749-8104-9-18-S2.png]

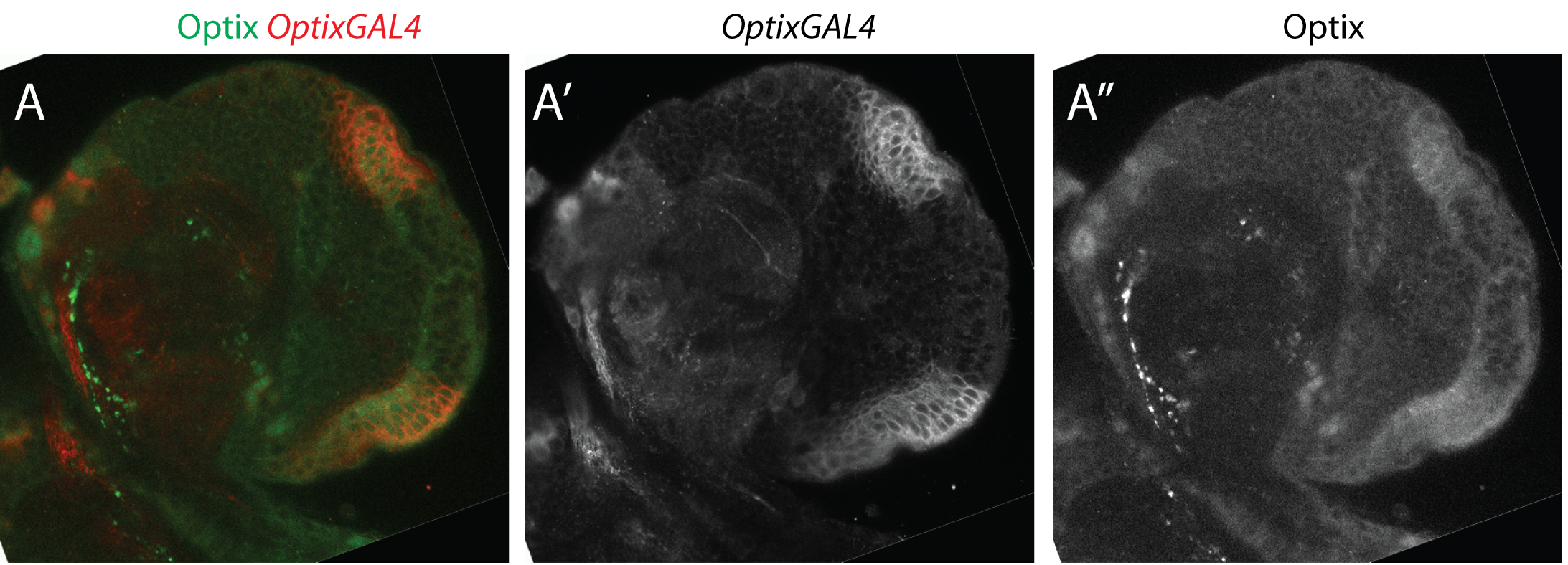

Supplement: Additional file 3 — OptixGAL4 recapitulates Optix protein expression. (A-A”) Posterior frontal cross-section of the optic lobe. OptixGAL4 driving UAS-mCD8GFP (red) showed a similarly well-defined expression pattern in the neuroepithelium to Optix protein (green). Both protein and the GAL4 line have sharp expression boundaries in the OPC neuroepithelium. [file 1749-8104-9-18-S3.png]

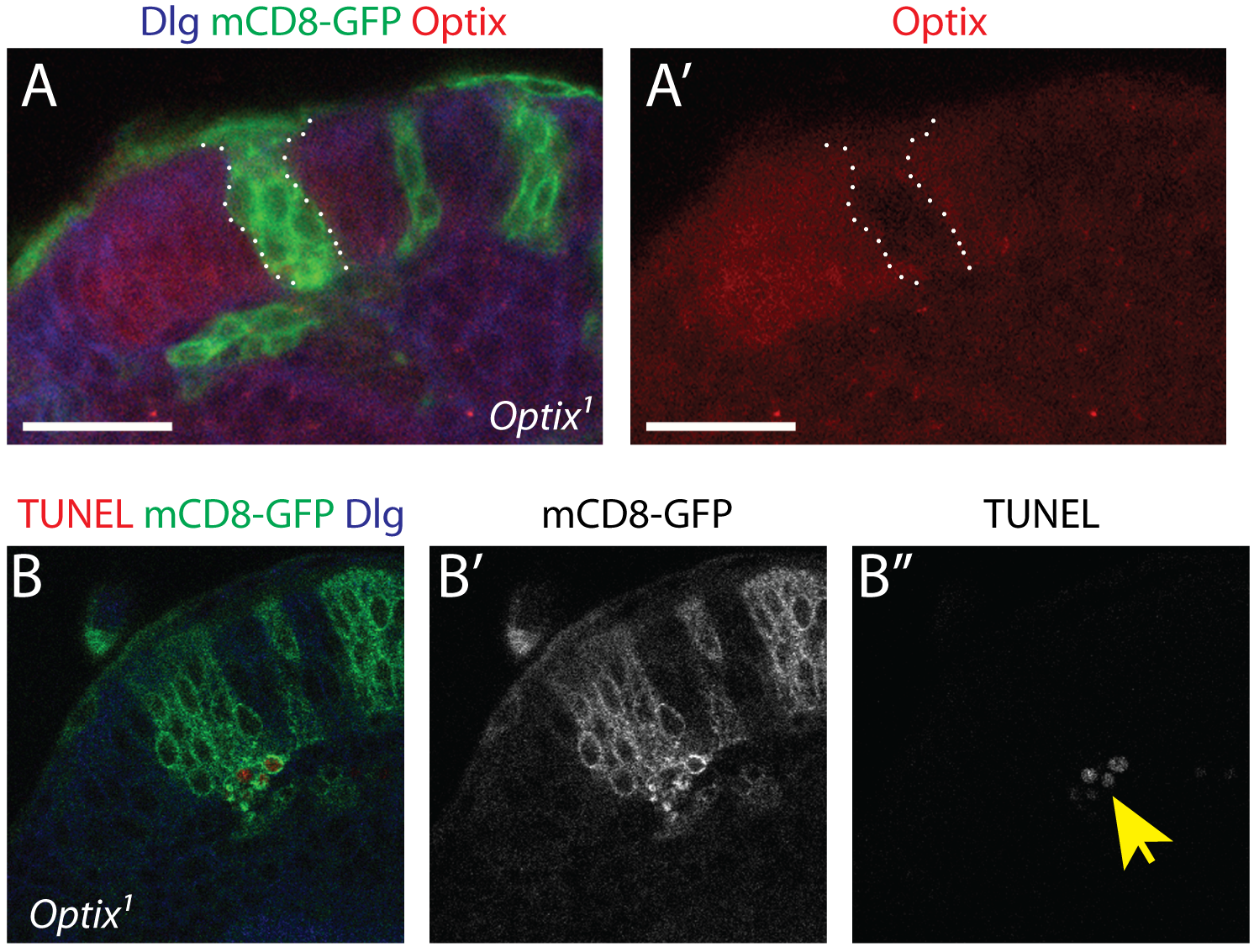

Supplement: Additional file 4 — Optix null mutant clones do not express Optix protein and undergo apoptosis. Description: Optix 1 mutant MARCM clones were induced in the neuroepithelium (labelled with mCD8-GFP in green, Dlg in blue). (A-A’) Optix 1 clones do not stain for Optix protein (red), indicating that they are null mutant clones. (B-B”) Optix 1 clones in the neuroepithelium undergo apoptosis. They are basally extruded from the neuroepithelium and stain positively for TUNEL (yellow arrow). [file 1749-8104-9-18-S4.png]

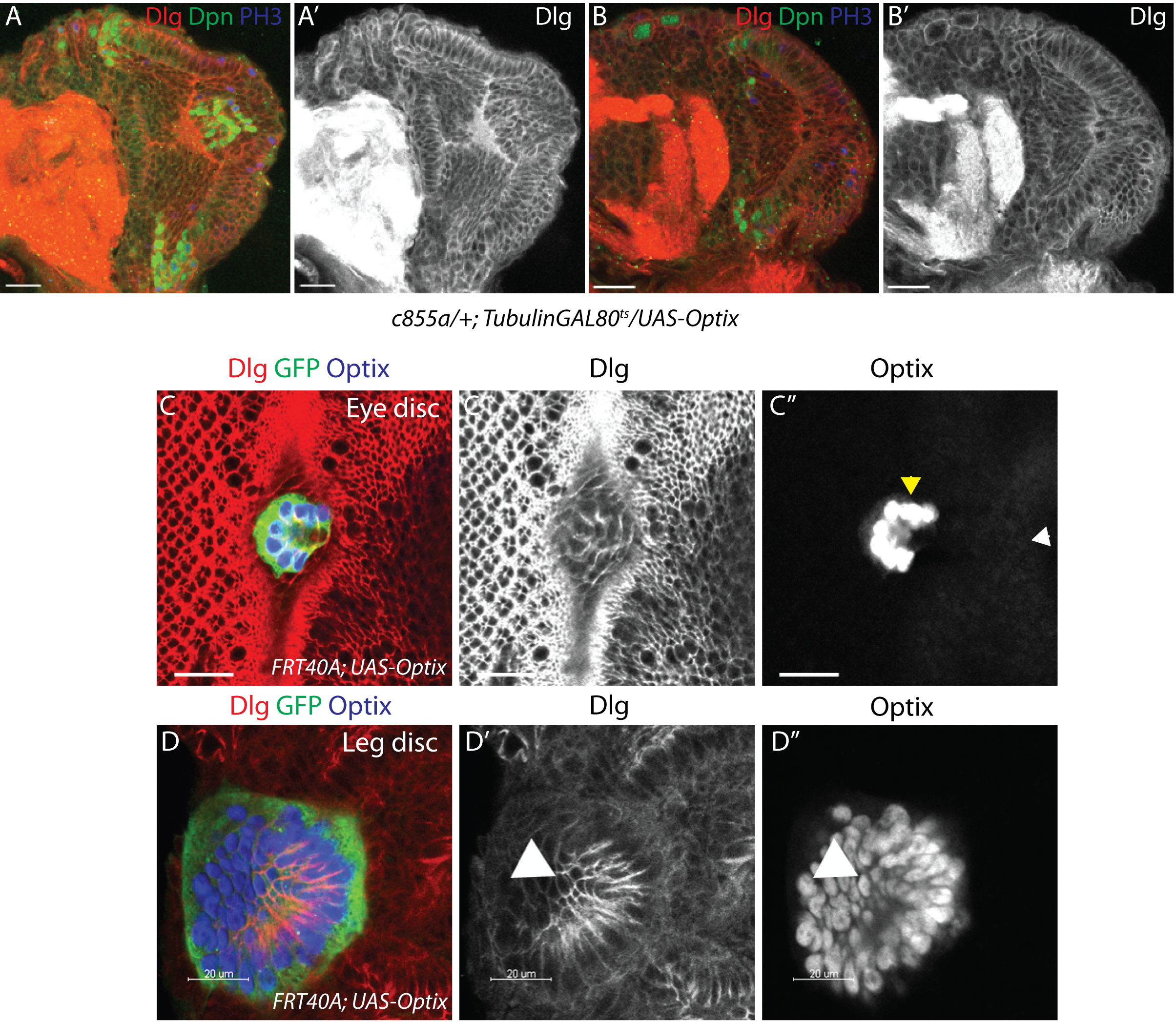

Supplement: Additional file 5 — Optix misexpression induces multilayering and clonal cell sorting. (A-B’) Optix misexpression throughout the neuroepithelium (with c855aGAL4) induces multilayering. Neuroepithelial cells take on a more rounded appearance, as opposed to their wild type columnar morphology. Cells are outlined by Dlg (red), neuroblasts stained by Dpn (green) and mitotic cells labelled by phospho-histone-H3 (PH3, blue). (C-D”) Optix misexpression clones induced in imaginal eye (C) and leg (D) discs form epithelial rosettes. Cells are outlined by Discs large staining (Dlg, red), clones labelled with mCD8-GFP (GFP, green), Optix stained in blue. (C”) Upon misexpression, Optix protein levels are very high (yellow arrowhead) compared to endogenous levels (white arrowhead). Misexpression clones sort away from their neighbours, and apical constriction and increased apical accumulation of Dlg protein (white arrowhead in D’) is visible. Scale bars: 20 μm. [file 1749-8104-9-18-S5.png]
